# Supplementary material for: Lunar rock investigation and tri-aspect characterization of lunar farside regolith by a digital twin
Source: Nat Commun. 2024 Mar 8;15:2098. doi: 10.1038/s41467-024-46233-8 (PMC11258293; doi:10.1038/s41467-024-46233-8)
Supplement: Supplementary file 1 — Supplementary Information [file 41467_2024_46233_MOESM1_ESM.pdf]

Supplementary Information for

Lunar rock investigation and tri-aspect characterization of lunar far side regolith by a digital twin

Liang Ding[1]*†, Ruyi Zhou[1]†, Tianyi Yu[2]†, Huaiguang Yang[1], Ximing He[2], Haibo Gao[1]*, Juntao Wang[3,4], Ye Yuan[1], Jia Wang[2], Zhengyin Wang[1], Huanan Qi[1], Jian Li[2]*, Wenhao Feng[1], Xin Li[2], Chuankai Liu[2,5], Shaojin Han[2], Xiaojia Zeng[3,4], Yu-Yan Sara Zhao[6], Guangjun Liu[7], Wenhui Wan[8], Yuedong Zhang[2], Saijin Wang[2], Lichun Li[2], Zongquan Deng[1], Jianzhong Liu[3,4]*, Guolin Hu[2], Rui Zhao[2], Kuan Zhang[2]

[1]*State Key Laboratory of Robotics and System, Harbin Institute of Technology; Harbin 150080, China.*

[2]*Beijing Aerospace Control Center; Beijing 100094, China.*

[3]*Center for Lunar and Planetary Sciences, Institute of Geochemistry, Chinese Academy of Science; Guiyang 550081, China.*

[4]*CAS Center for Excellence in Comparative Planetology; Hefei 230026, China.*

[5]*Key Laboratory of Science and Technology on Aerospace Flight Dynamics; Beijing 100049, China.*

[6]*Research Center for Planetary Science, College of Earth Science, Chengdu University of Technology; Chengdu 610059, China.*

[7]*Department of Aerospace Engineering, Ryerson University; Toronto, ON M5B 2K3, Canada.*

[8]*State Key Laboratory of Remote Sensing Science, Aerospace Information Research Institute, Chinese Academy of Sciences; Beijing 100101, China.*

**Corresponding author. Email: liangding@hit.edu.cn (Liang Ding), gaohaibo@hit.edu.cn (Haibo Gao), lisirjian@163.com (Jian Li) and liujianzhong@mail.gyig.ac.cn (Jianzhong Liu).*

†These authors contributed equally to this work: Liang Ding, Ruyi Zhou, Tianyi Yu

This PDF file includes:

Supplementary Notes

Supplementary Figure 1 to 13

Supplementary Table 1 to 13

Supplementary References

Supplementary Notes Nomenclature

A_t, B_t, C_t	normal vector of the wheel-terrain contact plane
$\boldsymbol{A}_1$, $\boldsymbol{A}_2$, $\boldsymbol{A}_3$	node of the triangular terrain mesh
b	wheel width
c	cohesion of the soil
c_1, c_2	coefficients of the wheel-terrain interaction angle
c_3	coefficient of the exit angle
f_{DP}	resistance force
F_{N}	normal force
F_{DP}	drawbar pull
F_{L}	lateral force
F_u	force beneath the wheel due to shear motion
F_{s}	resistance force acting on the side face of the wheel
$\boldsymbol{F}_e$	vector of equivalent forces in the initial frame
$^{e}\boldsymbol{F}_e$	vector of external forces acting on the wheel center
h	grouser height
j	shearing deformation of the soil
j_x	longitudinal shearing deformation of the soil (the soil deformation modulus in x direction)
j_y	lateral shearing deformation of the soil (the soil deformation modulus in y direction)
k_c	cohesive modulus of the soil
k_φ	frictional modulus of the soil
K_{s}	equivalent stiffness modulus of the soil
K_x	longitudinal shearing deformation modulus of the soil
K_y	lateral shearing deformation modulus of the soil
M_{R}	driving resistance torque
M_{S}	steering resistance torque
M_{O}	overturning torque
$\boldsymbol{M}_e$	vector of equivalent torques in the initial frame
$^{e}\boldsymbol{M}_e$	vector of external torques acting on the wheel center
n_0	static sinkage exponent
n_1	dynamic sinkage exponent
N	variable sinkage exponent of the wheel-terrain interaction
$\boldsymbol{p}_c$	position of the wheel center
$\boldsymbol{P}_1$, $\boldsymbol{P}_2$, $\boldsymbol{P}_3$, $\boldsymbol{P}_i$	coordinate point
r	wheel radius
r_{s}	equivalent shearing radius of a grouser wheel
$\boldsymbol{R}_e$	rotation matrix of the wheel-terrain interaction frame relative to the initial frame
s	slip ratio
T	driving torque
$\boldsymbol{T}_c$	homogeneous transform matrix of the wheel-terrain interaction frame relative to the initial frame
v	forward linear velocity
v_x	longitudinal travelling velocity
v_y	lateral travelling velocity
W	wheel load

z	wheel sinkage
β	side slip angle
θ_1	entrance angle
θ_2	exit angle
θ_i	solar incidence angle
θ_{m}	maximum stress angle
θ_1'	equivalent entrance angle of a grouser wheel
λ	wavelength
λ_{s}	lug shearing coefficient
σ	normal stress
σ_{m}	maximum normal stress
τ	shear stress
τ_x	longitudinal shear stress
$\tau_{x\mathrm{m}}$	maximum longitudinal shear stress
τ_y	lateral shear stress
$\tau_{y\mathrm{m}}$	maximum lateral shear stress
φ	internal friction angle of the soil
φ_y	external friction angle of the soil
ω	angular velocity
Σ_I	inertial frame
Σ_e	wheel-terrain interaction frame
Σ_c	wheel center frame

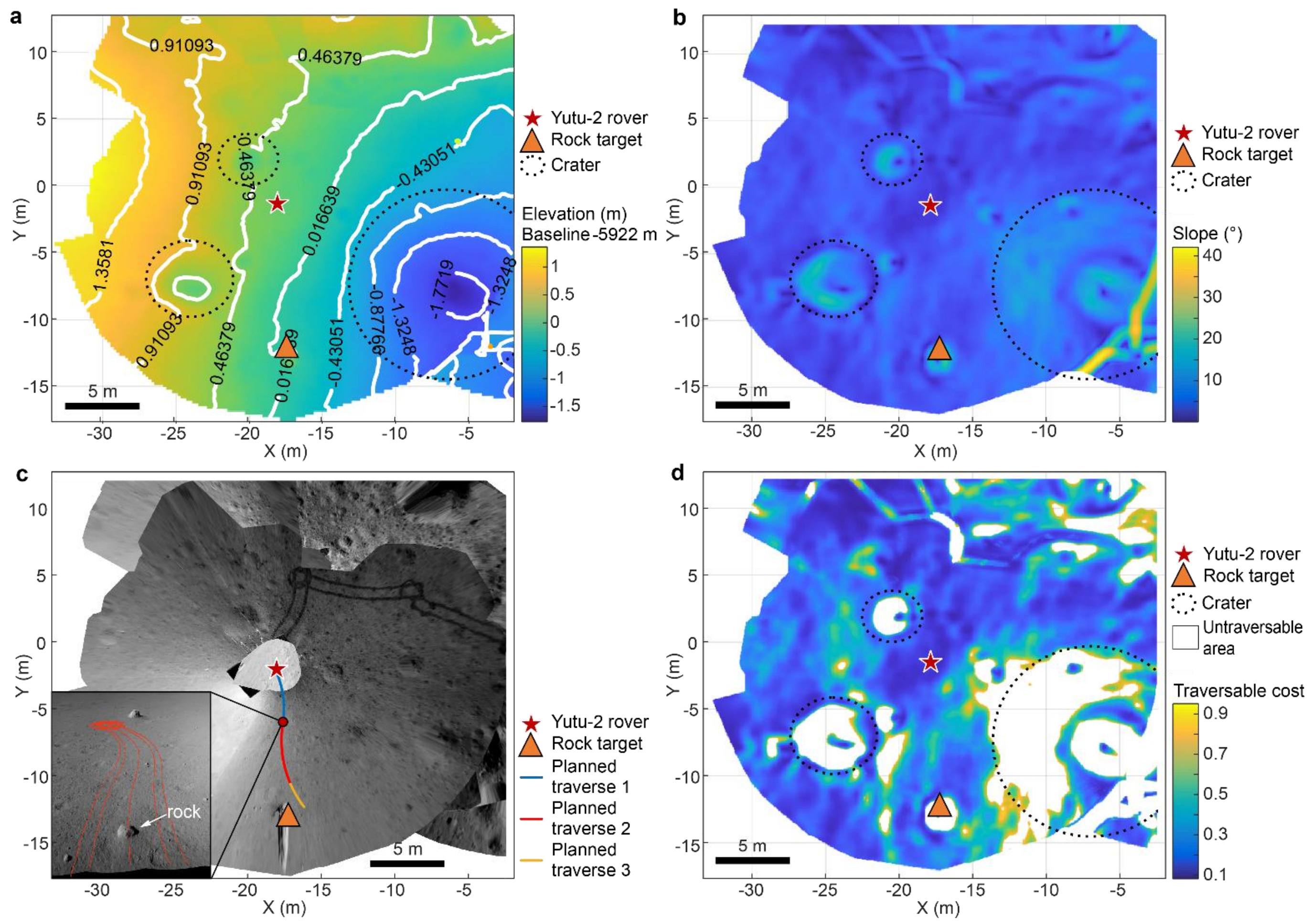

Supplementary Fig. 1 Local terrain analysis around the Longji site. a, Counter plot of the terrain elevation around the Longji site. **b**, Slope map of the Longji site. **c**, Digital orthophoto map around the Longji site with planned traverses to the target rock. The inset is a Pancam image taken on the 40th lunar day showing a small rock on the approaching path to the target rock. **d**, Traversable cost map of the Longji site. The traversable cost is a dimensionless quantity ranging from 0.0 to 1.0. Higher traversable cost means risker, while lower traversable cost means safter to traverse. The traversable cost is determined by slope steepness, terrain roughness, step height, and other factors.

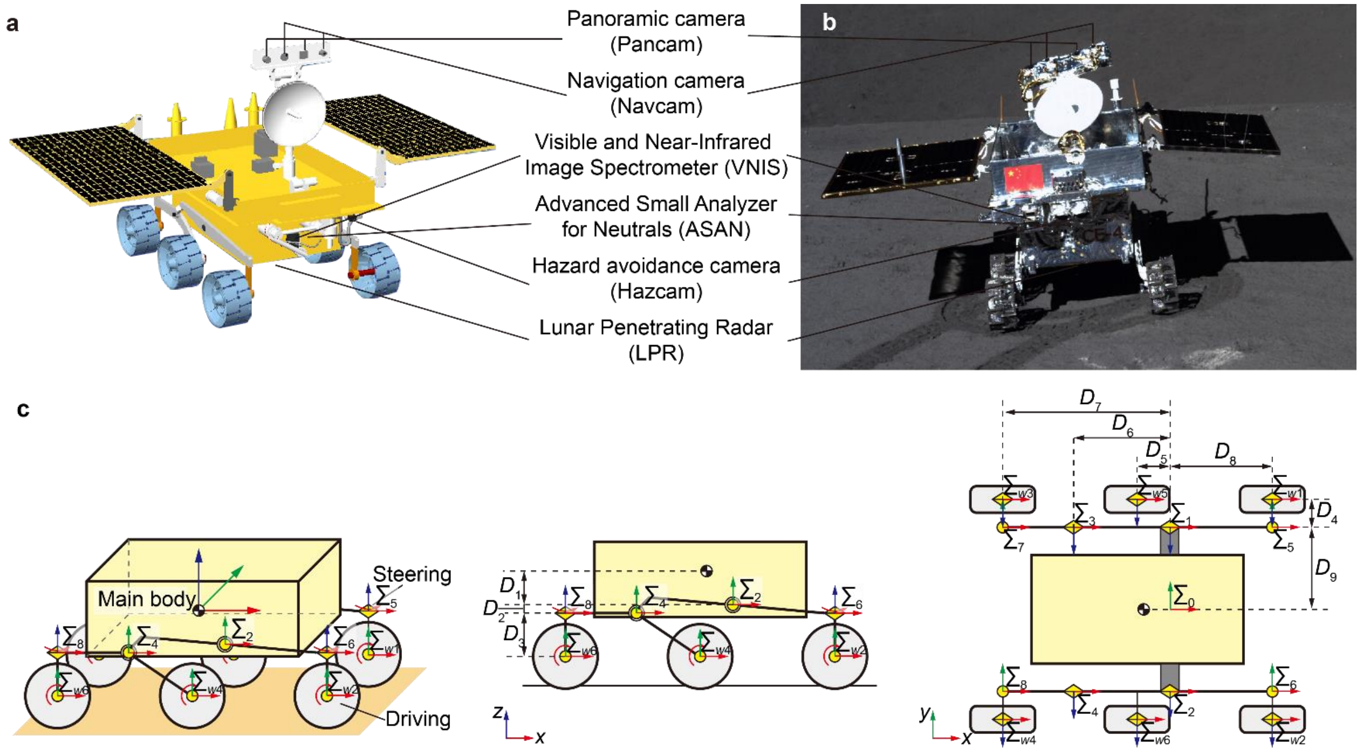

Supplementary Fig. 2 Yutu-2 rover model. **a**, Virtual Yutu-2 computer aided design (CAD) model in the digital space. **b**, Yutu-2 rover in the physical space. The raw image was from: https://rb.gy/7lloxg. **c**, Dynamic model of the Yutu-2 rover with the associated coordinate systems. $\{\sum_0\}$ is the base coordinate system of the rover; its *x*-axis and *y*-axis are parallel to the longitudinal and lateral direction of the rover respectively, and its *z*-axis is vertical to them. $\{\sum_i\}$ (i=1,2,3,..,8) and $\{\sum_{wj}\}$ (j=1,2,3,..,6) are inertial coordinate systems on joints or axles. All of these coordinate systems are right-hand frames. Detailed variables of defining these coordinate systems are listed in Supplementary Table 10.

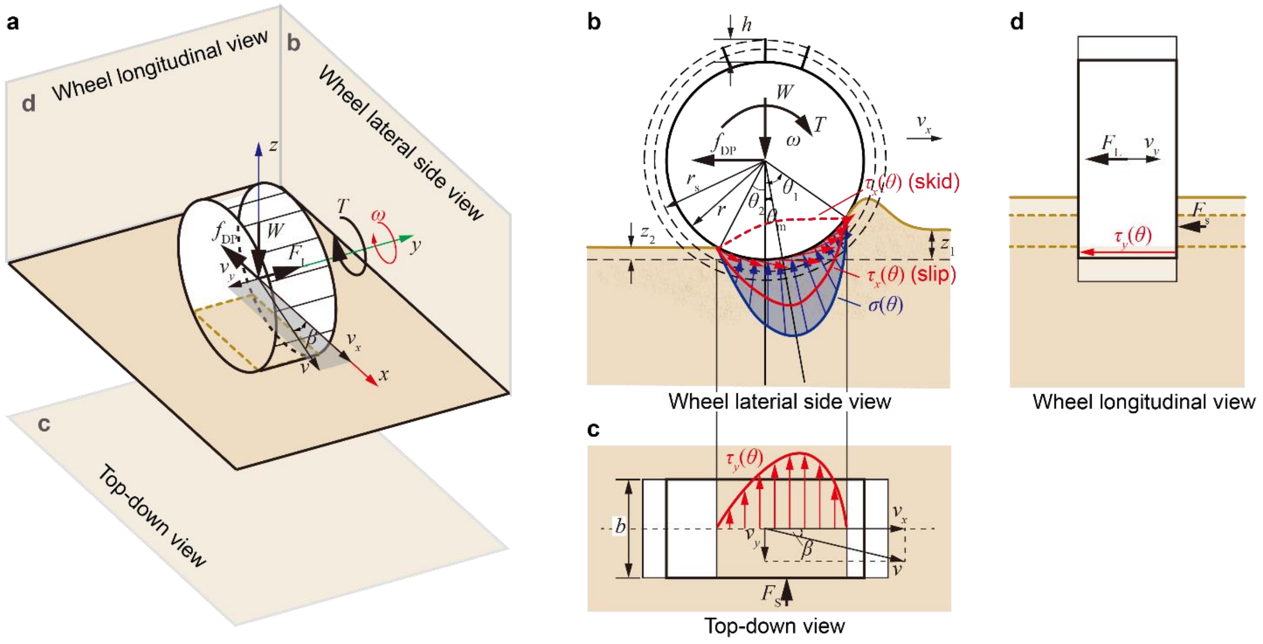

Supplementary Fig. 3 Stress distribution of the wheel-terrain interaction for a grouser wheel moving on soft and deformable terrain with a longitudinal skid. **a**, Schematic diagram of a grouser wheel moving on soft and deformable terrain with side slip angle. **b**, Normal stress and shear stress distribution from the wheel lateral side view. z_1 is the wheel sinkage; z_2 is the soil rebound; The red solid line is the envelope curve of $\tau_x(\theta)$ when the wheel is in the slip state ($0 \leq s < 1$), while the red dashed line is the envelope curve of $\tau_x(\theta)$ when the wheel is in the skid ($-1 < s < 0$) state. **c**, Shear stress distribution from the top-down view. **d**, Shear stress distribution from the wheel longitudinal view. F_s is the resistance force acting on the side face of the wheel.

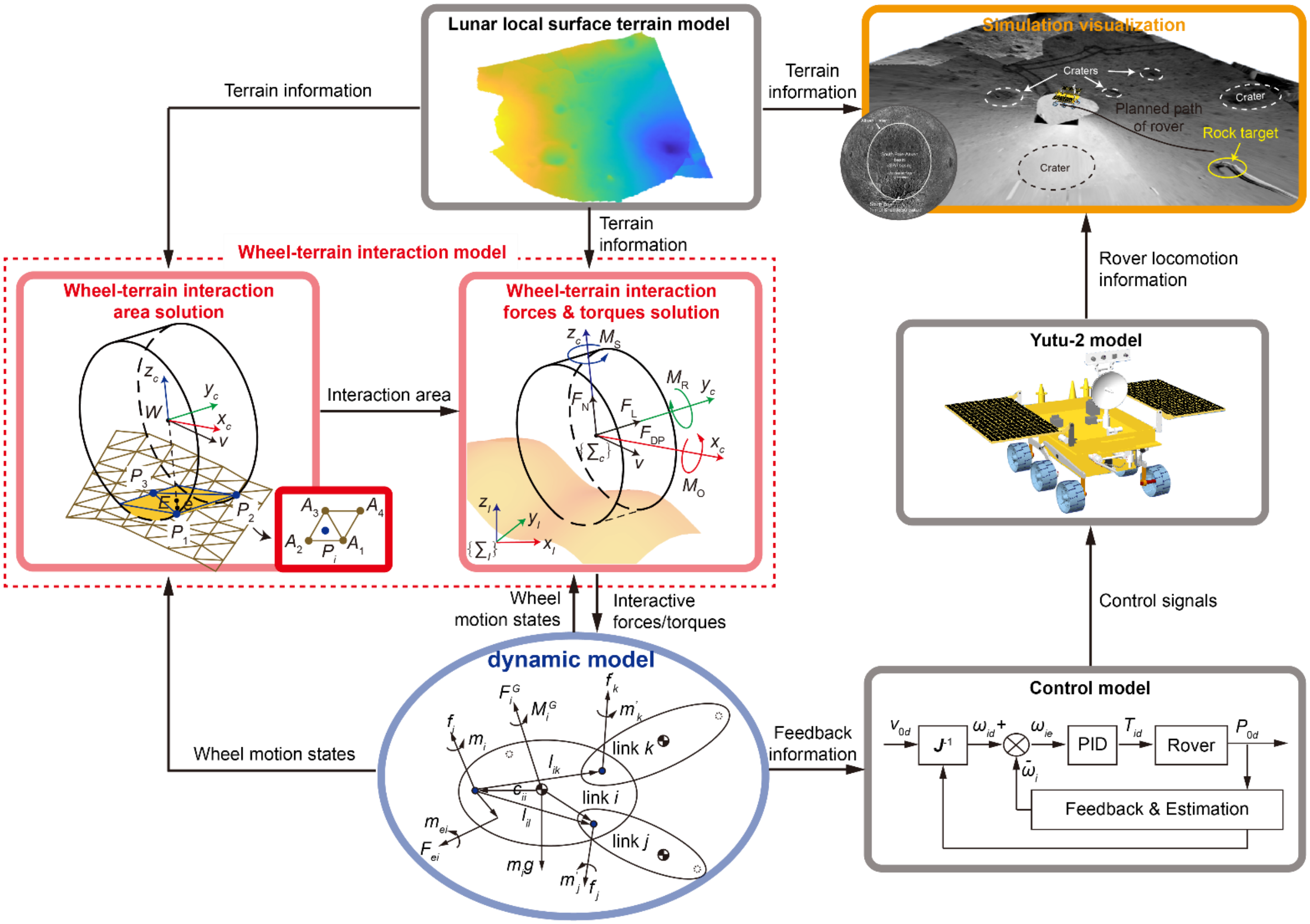

Supplementary Fig. 4 Diagram of the rover dynamic simulation. At the simulation onset, the lunar local surface terrain model and Yutu-2's virtual computer aided design (CAD) model in gray boxes are loaded, alongside the pre-designed rover control model (in gray box). The core of the dynamic simulation is the wheel-terrain interaction model (highlighted in red dotted box), which takes terrain information and wheel motion states as inputs, solving for the wheel-terrain interaction area and computing interactive forces and torques. This model is composed of two parts: the wheel-terrain interaction area solver, and the wheel-terrain interaction forces and torques solver. This former module calculates two sets of parameters based on wheel position and velocity: terrain mechanical parameters of the interaction area defined by interaction points $\boldsymbol{P}_1$, $\boldsymbol{P}_2$, $\boldsymbol{P}_3$, and key wheel motion states (wheel sinkage z, the slip ratio s, and the side-slip angle β). These data are fed into the latter module (wheel-terrain interaction force and torque solver), which outputs interactive forces and torques between the wheel and terrain in three directions (F_{N}, F_{DP}, F_{L}, M_{S}, M_{O}, M_{R}) using a terramechanics model. With obtained data and control commands, the rover's locomotion information including position, velocity, and acceleration are updated by solving dynamics with rover's dynamic model (in blue box). The lunar surface terrain model and updated rover states are visualized in the display interface (in orange box).

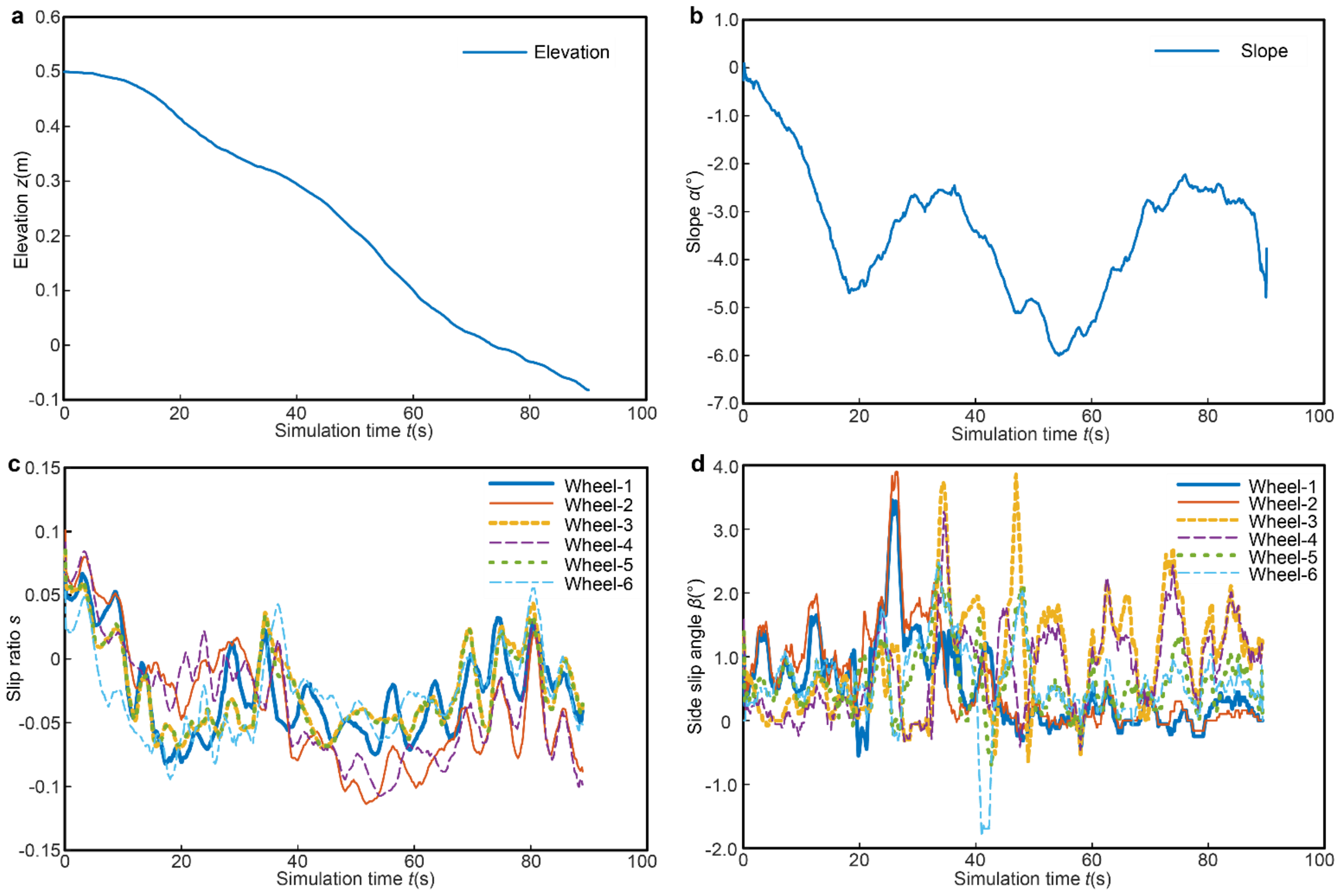

Supplementary Fig. 5 Simulation results of the outboard traverse conditioned on φ=25°, K_y=40 mm. Data is collected at 10 Hz. **a**, Terrain elevation of the outboard traverse. **b**, Terrain slope of the outboard traverse. **c**, Slip ratio of six wheels during the outboard traverse. The slip ratio data is further smoothed with 20 points using the adjacent-averaging filter. **d**, Side slip angle of six wheels during the outboard traverse. The side slip angle data is further smoothed with 20 points using the adjacent-averaging filter. The filtered data are provided as a Source Data file.

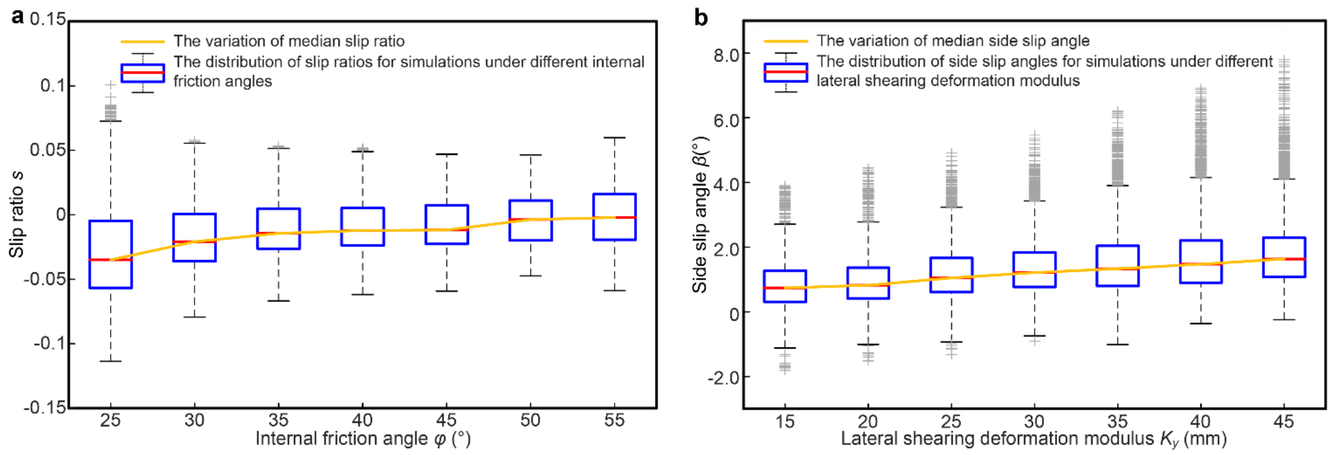

Supplementary Fig. 6 Wheel state analysis of the cluster of predicted traverses. The data is from six wheels and further smoothed with 20 points using the adjacent-averaging filter. **a**, Wheel slip ratios of predicted traverses under different internal friction angles. Each slip ratio distribution is defined by the minimum (lower black line), the first quartile (lower blue line), the median (middle red line), the third quartile (upper blue line), the maximum (upper black line) from bottom to the top. Gray crosses are outliers of each distribution. **b**, Wheel side slip angles of the predicted traverse under different lateral shearing deformation modulus. Each side slip angle distribution is defined in the same way subfigure a. Gray crosses are outliers of each distribution. The filtered data are provided as a Source Data file.

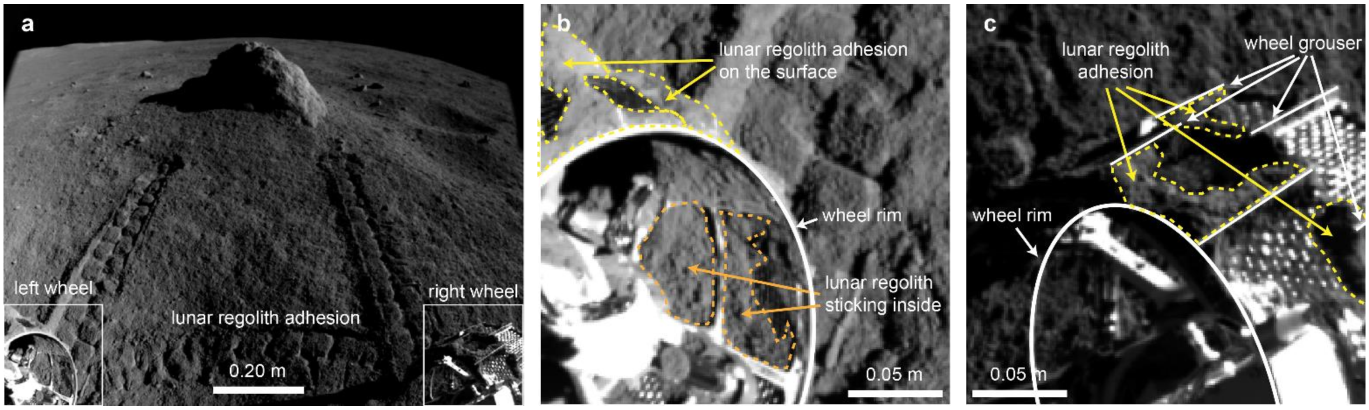

Supplementary Fig. 7 Lunar regolith sticky phenomenon of the Yutu-2 rover. a, Hazcam image showing lunar regolith adhesion on both the surface of the left and the right wheel. The image was taken on the 41st lunar day. **b**, Enlarged view of the left regolith adhesive wheel. Fine-grained lunar soil sticks on the surface. Some regolith even entered and adhered to the inside of the wire mesh wheel after crushed. **c**, Enlarged view of the right regolith adhesive wheel. Lunar regolith sticks around the wheel rim and the grousers.

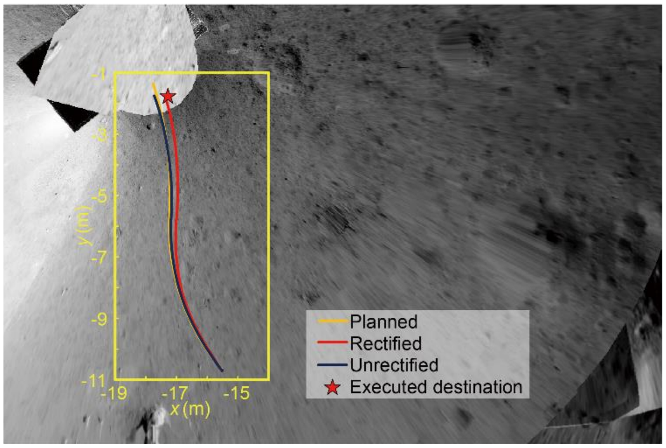

Supplementary Fig. 8 Yutu-2's backhaul prediction with rectified regolith parameters compared with the planned route. All the trajectories start from the same point, where the rover stop to do rock investigation. The rectified trajectory is predicted using parameters estimated based on the outboard journey, while the unrectified trajectory is predicted using typical parameters. The parameters for prediction the rectified and unrectified trajectory are list in Supplementary Table 8.

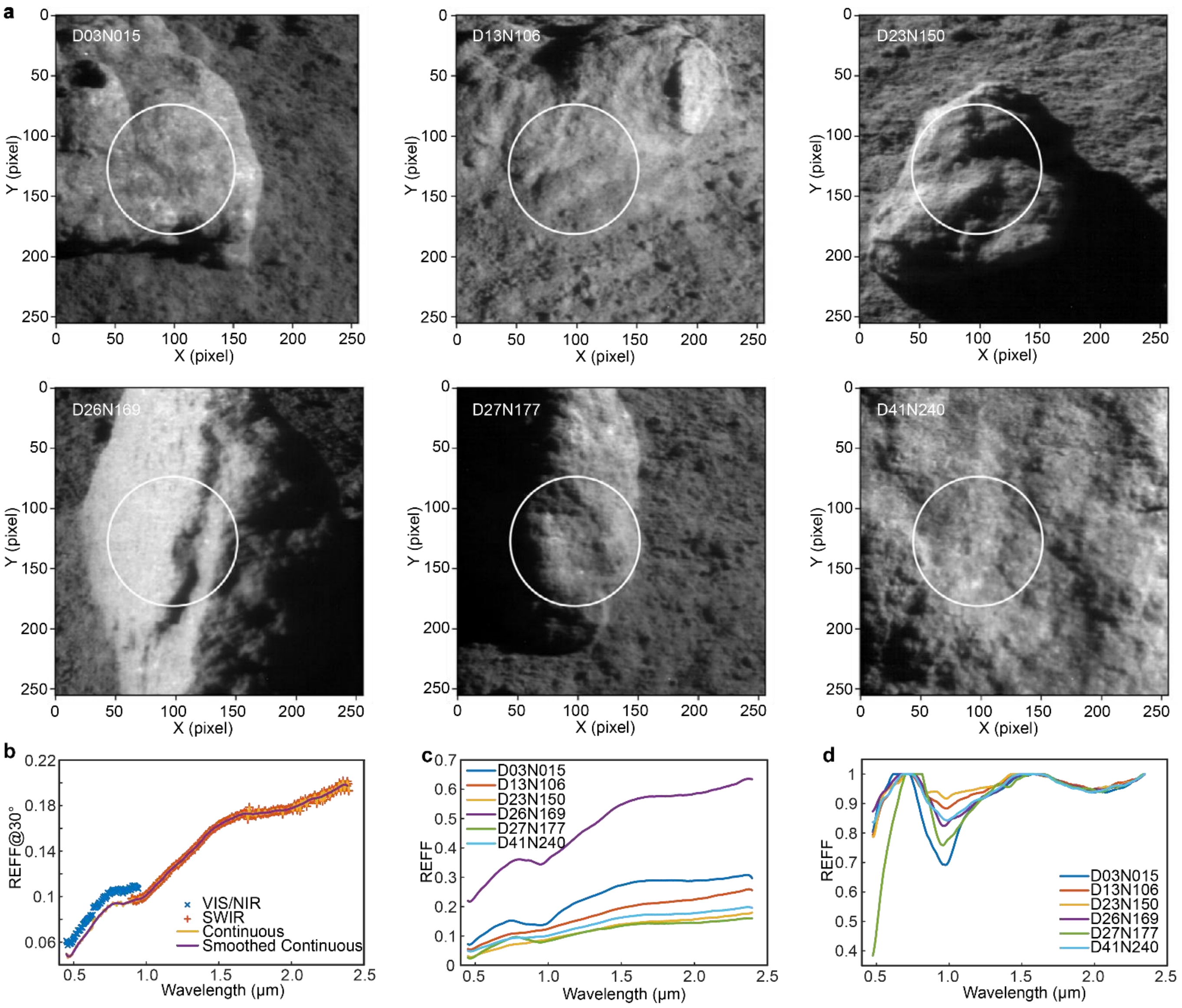

Supplementary Fig. 9 Images and spectra of six rocks investigated along the Yutu-2 rover traverse. a, Images of six rocks detected in the 750 nm band in the VNIS. DXXNXXX means the detected points NXXX on the lunar-day DXX. **b**, Observed spectra of the target boulder in the origin, continued, smoothed and envelope lines. VIS/NIR and SWIR illustrate the spectrum discontinuity. The envelope line is the conic line fitted by three points at 750, 1580 and 2345 nm. **c**, Six rock spectra after smoothing. **d**, Continuum-removed spectrum for six rocks. Source data are provided as a Source Data file.

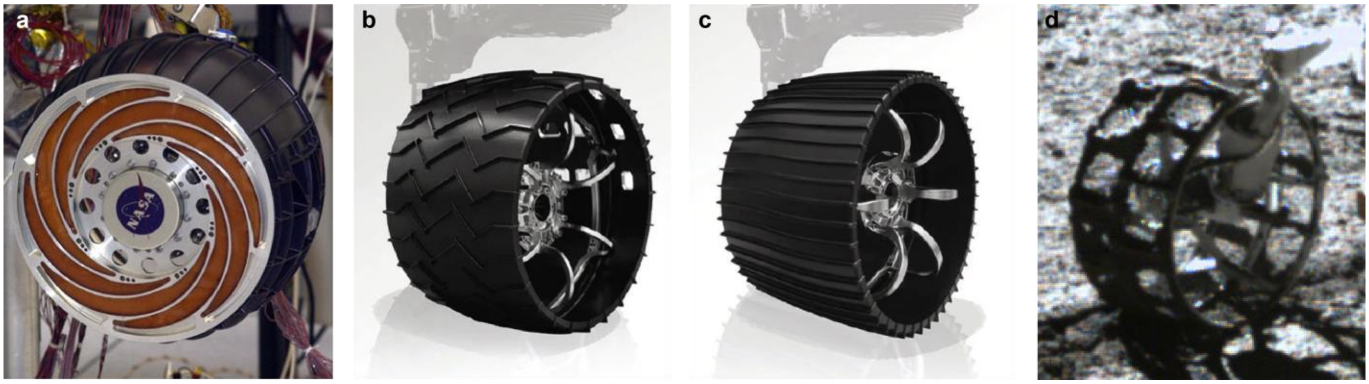

Supplementary Fig. 10 Comparison of rover wheels. **a**, Wheel of Mars Exploration rover (MER). The wheel is 0.25 m in diameter. The source image is from: https://rb.gy/xi4x7o. Credit: Courtesy NASA/JPL-Caltech. **b**, Wheel of Curiosity rover in Mars Science Laboratory (MSL) mission. The wheel is 0.508 m in diameter. The source image is from: https://rb.gy/uw2mh2. Credit: Courtesy NASA/JPL-Caltech. **c**, Wheel of Perseverance rover in Mars 2020 mission. The wheel is 0.526 m in diameter. The source image is from: https://rb.gy/uw2mh2. Credit: Courtesy NASA/JPL-Caltech. **d**, Wheel of Yutu-2 rover in Chang'E-4 mission. The wheel is 0.3 m in diameter. The raw image was available in lunar and planetary data release system of China at: https://rb.gy/l9gb2f. Credit: CNSA/CAS.

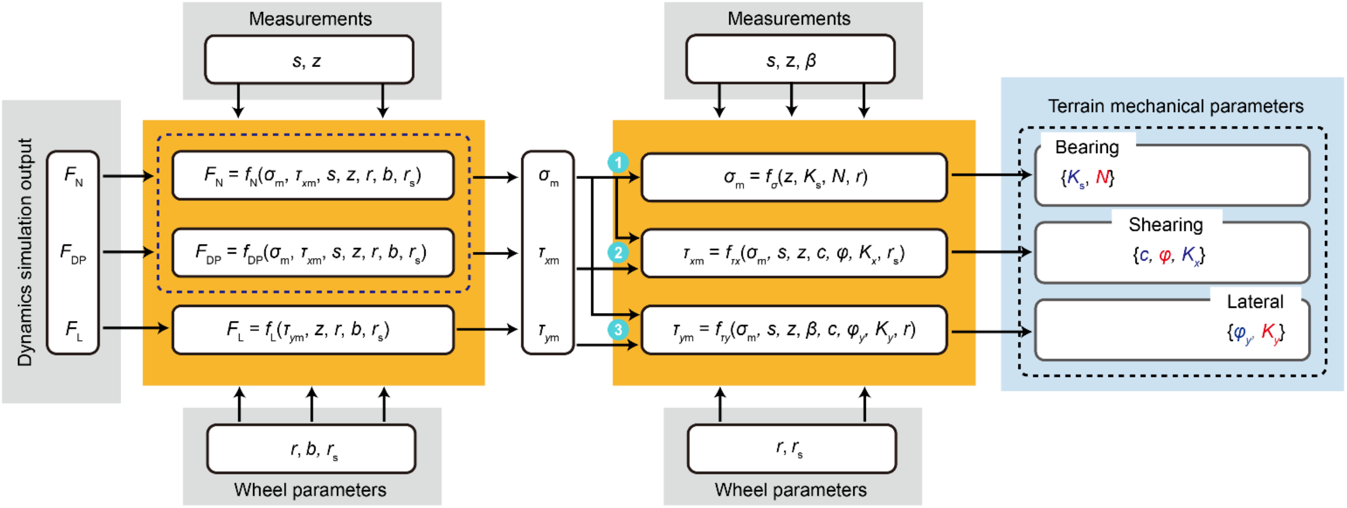

Supplementary Fig. 11 Regolith parameter estimation. Parameters in gray box are input of the framework, and they are fixed parameters set according to wheel parameters or obtained by the dynamic solution or external measurements. Equations in the orange boxes are key equations for parameter identification. Parameters in blue box are the output of the framework and are specific to terrain bearing, shearing and lateral parameters. The terrain mechanical parameters in bearing, shearing and lateral aspects are categorized into dominant (in red) and non-dominant parameters (in blue). Acting as pivotal links, the maximum normal stress σ_m, the maximum longitudinal shear stress τ_{xm}, and the maximum lateral shear stress τ_{ym} are firstly solved. Leveraging inputs of wheel forces (F_N, F_{DP}), wheel motion states (slip ratio s, sinkage z), and fixed wheel parameters (r, b, r_s), σ_m and τ_{xm} are jointly determined by equations involving F_N and F_{DP}. τ_{ym} is independently calculated using the equation of lateral force F_L. Using only σ_m, represented in $\{z, K_s, N, r\}$, the bearing property relationship between sinkage exponent N and equivalent stiffness modulus K_s is revealed. Further, the relationship between shearing and lateral parameters are unveiled by combining σ_m with τ_{xm} and τ_{ym}, respectively.

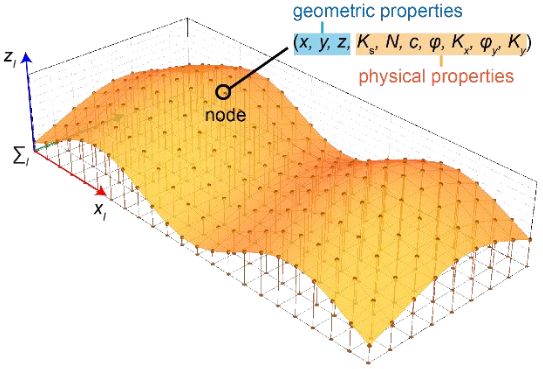

Supplementary Fig. 12 Description of the DEMP^2 used in the dynamic simulation. The DEMP^2 is separated into discrete nodes and each node has its own geometric and physical properties. The geometric properties are represented in 3-dimensional position of the node in inertial frame as (x, y, z). The physical properties of each node are represented as (K_s, N, c, φ, K_x, φ_y, K_y). K_s is the equivalent stiffness modulus of the soil, N is the sinkage exponent, c is the cohesion of the soil, φ is the internal friction angle of the soil, K_x is the longitudinal shearing deformation modulus of the soil, φ_y is the external friction angle of the soil, K_y is the lateral shearing deformation modulus of the soil.

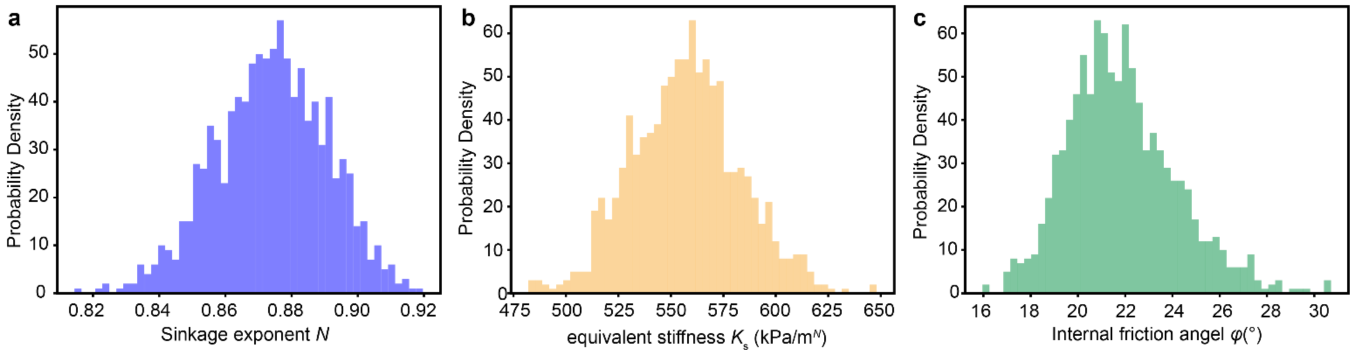

Supplementary Fig. 13 Uncertainty of the estimated mechanical properties using Monte Carlo simulations. a, Distribution of estimated sinkage exponents considering the measurement error of wheel sinkage and interaction forces. The results are conditioned on wheel sinkage of 5 mm with the standard deviation of 0.33 mm, and normal force of 32.027 N with 10% uncertainty, and drawbar pull of -1.33 N with 10% uncertainty. The mean of the sinkage exponent is 0.87 with standard deviation of 0.017. **b,** Distribution of estimated equivalent stiffness considering the measurement error of wheel sinkage and interaction forces. The results are conditioned on wheel sinkage of 15 mm with standard deviation of 0.33 mm, and the same force error as (**a**). **c,** Distribution of estimated internal friction angles considering the measurement error of slip ratio and force uncertainty. The results are conditioned on slip ratio of -0.075 with 4% uncertainty. The forces are in the same setting as (**a**). The colors of these three subfigures are not associated to elements provided in other figures.

Supplementary Table 1 The planned path and associated parameters.

Phase		Movement type	Coordinate of the start point (m)	Rover heading direction at the start point	Coordinate of the end point (m)	Rover heading direction at the end point	Curvature (m^{-1})	Curve length (m)
Outbound journey	1	Ture in place	(-1.442, -17.871)	-90.718°	(-1.442, -17.871)	156.436°	-	
	1-2	Curve	(-1.442, -17.871)		(-5.036, -17.404)		0.154	3.674
	2-3	Curve	(-5.036, -17.404)		(-9.227, -16.862)		-0.132	4.282
	3-4	Curve	(-9.227, -16.862)		(-10.812, -15.914)		-0.138	1.852
	4	Ture in place	(-10.812, -15.914)	141.796°	(-10.812, -15.914)	-136.810°	-	
	4-5	Straight line	Move forward straightly				∞	
Return journey	5	Turn in place	(-10.847, -15.939)	-139.483°	(-10.847, -15.939)	-40.366°	-	-
	5-6	Curve	(-10.847, -15.939)		(-5.741, -17.655)		0.138	5.518
	6-7	Curve	(-5.741, -17.655)		(-1.504, -18.168)		-0.084	4.292
	7	Turn in place	(-1.504, -18.168)	-12.735°	(-1.5035, -18.168)	165.000°	-	-

Note: A positive curvature represents that the rover is moving and turning right, while a negative curvature represents a left turn of the rover. In the 1-2, 2-3, 3-4 sections, the rover moves according to settled constructions, while in the 4-5 section, it's movement to approach the rock target for investigation is pendent.

Supplementary Table 2 Normalized band strength ratio (NBSR) values of six rock spectra at the CE-4 site.

NBSR	1000 nm absorption			2000 nm absorption	
	Olivine	Orthopyroxene	Clinopyroxene	Orthopyroxene	Clinopyroxene
D03N015	25.56%	33.89%	40.54%	44.87%	55.13%
D13N106	37.68%	17.96%	44.36%	46.82%	53.18%
D23N150	12.89%	78.46%	8.65%	48.26%	51.74%
D26N169	21.24%	41.66%	37.10%	45.27%	54.73%
D27N177	18.69%	35.45%	45.87%	48.45%	51.55%
D41N240	30.42%	24.66%	44.92%	45.72%	54.28%

Note: DXXNXXX means the detected points NXXX on the lunar-day DXX.

Supplementary Table 3 The abundance of minerals from Kaguya Multiband Imager (MI) data.

Location	Latitude	Longitude	Plagioclase	Orthopyroxene	Clinopyroxene	Olivine
Finsen CP	-42.27	-177.71	45%	38.5%	16.5%	0%
Finsen Wall	-42.42	-179.20	55%	22.5%	18%	4.5%
Zhinyu ejecta	-45.28	176.35	34%	19.8%	33%	13.2%

Supplementary Table 4 The parameters of the Yutu-2 lunar rover.

Group	Parameter name	Value
Rover	Mass m (kg)	135
	Size $l \times w \times h$ (m)	1.5×1.1×1.0
	Number of wheels	6
	Maximum driving speed v_{max} (m h^{-1})	200
	Maximum climbable slope (°)	20
	Climbable obstacle (mm)	200
	Track (mm)	950
	Wheelbase (mm)	600
	Steering offset (mm)	106
Wheel	Wheel radius r (m)	0.15
	Wheel width b (m)	0.15
	Height of the wheel grouser h (m)	0.01
	Number of wheel grousers	24
	Equivalent shearing radius of a wheel r_s (m)	0.1565

Note: Six wheels on the Yutu-2 can drive, and only four corner wheels have steering motors. Track is the distance between the left and right wheels. Wheelbase is the distance between the centers of the front wheels and the middle wheels, or the distance between the centers of the middle wheels and the rear wheels. The distance between the centers of the front and middle wheels is the same as the that between the centers of the middle wheels and the rear wheels for Yutu-2 rover. The 24 wheel grousers are arranged in two staggered rows, and the grousers in each row are evenly arranged on the wheel's outer cylinder surface.

Supplementary Table 5 Major specifications of the Pancam[1], Navcam and Hazcam.

Camera	Parameter	Value
Pancam (stereo; two modes)	Baseline	270 mm
	Waveband	red: 640 nm, green: 540 nm, blue: 470 nm @ color mode<br>420-700 nm @ panchromatic mode
	Field of view	19.6°×14.5°
	Image resolution	2,352×1,728 pixels @ color mode<br>1,176×864 pixels @ panchromatic mode
	Resolving power	0.15 mrad (about 120 pixels $degree^{-1}$)
Navcam (stereo)	Baseline	270 mm
	Focal length	17.7 mm
	Field of view	46.6°
	Image resolution	1024×1024 pixels
Hazcam (stereo; fisheye camera)	Field of view	170°
	Image resolution	1024×1024 pixels

Note: The Pancam can acquire a 360° panoramic view by taking 56 pairs of images (28 different azimuth angles at 2 elevation angles).

Supplementary Table 6 Major specifications of the visible and near-infrared imaging spectrometer (VNIS)[2].

Description	Specification	
	VIS/NIR	SWIR
Spectral range (nm)	450-950	900-2400
Spectral resolution (nm)	2.4-6.5	3.6-9.6
Field of view (°)	8.5×8.5	Φ3.58
Bit resolution	256×256	1
Signal to noise ratio (dB)	>33	>31
Power consumption (W)	16.95	
Weight (kg)	4.65 (probe part)<br>0.7 (electronics part)	

Supplementary Table 7 Inputs values of the wheel-terrain interaction models for parameter identification.

Group	Variables (Unit)	Value
Forces	Normal force F_N (N)	32.02
	Drawbar pull F_{DP} (N)	-1.33
	Lateral force F_L (N)	-3.16
Wheel states	Slip ratio s	0 ~ -0.075
	Sinkage z (m)	0.005 ~ 0.015
	Side slip angle β (°)	0.5 ~ 3
Wheel parameters	Wheel radius r (m)	0.15
	Wheel width b (m)	0.15
	Equivalent shearing radius of a wheel r_s (m)	0.1565

Supplementary Table 8 Setting of the terrain mechanical parameters in the simulation.

Phase	Number of groups	K_s(kPa m^{-N})	N	c(Pa)	φ(°)	K_x(mm)	φ_y(°)	K_y(mm)
Outboard journey	7	827	1.0	520	25-55	17.8	0.3×φ	40
	7	827	1.0	520	40	17.8	0.3×φ	15-45
Return journey	1(unrectified)	827	1.0	520	42	17.8	12.6	20
	1(rectified)	827	0.9	520	38.95	17.8	12.6	40

Supplementary Table 9 Parameters of terrestrial soil samples.

Terrain	n	k_c(kPa m^{-N+1})	k_φ(kPa m^{-N})	K_s(kPa m^{-N})	c(kPa)	φ(°)
Dry sand (Land Locomotion Lab., LLL)	1.1	0.99	1528.43	1535.03	1.04	28
Sandy loam (LLL)	0.7	5.27	1515.04	1550.17	1.72	29
Sandy loam Michigan (Strong, Buchele)	0.9	52.53	1127.97	1478.17	4.83	20
Sandy loam (Hanamoto)	0.3	2.79	141.11	159.71	13.79	22
Clayey soil (Thailand)	0.5	13.19	692.15	780.08	4.14	13
Lean clay (WES)	0.2	16.43	1724.69	1834.22	68.95	20
LETE sand (Wong)	0.79	102	5301	5981	1.3	31.1
Upland sandy loam (Wong)	1.1	74.6	2080	2577	3.3	33.7
Rubicon sandy loam (Wong)	0.66	6.9	752	798	3.7	29.8
North Gower clayey loam (Wong)	0.73	41.6	2471	2748	6.1	26.6
Grenville loam (Wong)	1.01	0.06	5880	5880	3.1	29.8
Snow (U.S.)	1.6	4.37	196.72	225.85	1.03	19.7
Snow (Sweden)	1.44	10.55	66.08	136.41	6	20.7

Note: n, k_c, k_φ, c, φ are original data from reference[3], while the values of K_s for terrains are derived based on k_c and k_φ when the wheel width is 0.15 m. Previously, the dynamic sinkage caused by slip ratio is not considered; thus, it is deemed that N=n, where n is the intrinsic sinkage exponent of the terrain, and N is the variable sinkage exponent of the wheel-terrain interaction.

Supplementary Table 10 Parameters of lunar soil obtained in other lunar exploration missions[4].

Mission (year)	Landing site	Identification method	c(kPa)	φ(°)
Apollo 11 (1969)	Mare Tranquilliatis	Penetrometer	0.25-0.85	38-42
Apollo 12 (1969)	Ocean of Storms	Vacuum Direct Shear	0-0.7	28-35
		Direct Shear (Surveyor 3)	0.1-3.1	13-56
		Triaxial Shear (Surveyor 3)	0-1	51-59
Luna 16 (1970)	Mare Fecunditatis	Direct Shear and Coulomb Device	3.9-5.9	20-25
Luna 20 (1972)	Apollonius Hightlands			
Lunokhod 2 (1973)	Le Monnier Crater	Estimated	0.4	40

Supplementary Table 11 Kinematics parameters of Yutu-2 rover.

Coordinate	Actuated	X axis (m)	Y axis (m)	Z axis (m)	θ_x	θ_y	θ_z
$\{\sum_0\}\rightarrow\{\sum_1\}$	×	0	D_9	$-D_1$	0	θ_1	0
$\{\sum_0\}\rightarrow\{\sum_2\}$	×	0	$-D_9$	$-D_1$	0	θ_2	0
$\{\sum_1\}\rightarrow\{\sum_3\}$	×	$-D_6$	0	$-D_2$	0	θ_3	0
$\{\sum_2\}\rightarrow\{\sum_4\}$	×	$-D_6$	0	$-D_2$	0	θ_4	0
$\{\sum_1\}\rightarrow\{\sum_5\}$	✓	D_8	0	$-D_2$	0	0	θ_5
$\{\sum_2\}\rightarrow\{\sum_6\}$	✓	D_8	0	$-D_2$	0	0	θ_6
$\{\sum_1\}\rightarrow\{\sum_7\}$	✓	$-D_7$	0	$-D_2$	0	0	θ_7
$\{\sum_2\}\rightarrow\{\sum_8\}$	✓	$-D_7$	0	$-D_2$	0	0	θ_8
$\{\sum_5\}\rightarrow\{\sum_{w1}\}$	✓	0	D_4	$-D_3+D_2$	0	θ_9	0
$\{\sum_6\}\rightarrow\{\sum_{w2}\}$	✓	0	$-D_4$	$-D_3+D_2$	0	θ_{10}	0
$\{\sum_7\}\rightarrow\{\sum_{w3}\}$	✓	0	D_4	$-D_3+D_2$	0	θ_{11}	0
$\{\sum_8\}\rightarrow\{\sum_{w4}\}$	✓	0	$-D_4$	$-D_3+D_2$	0	θ_{12}	0
$\{\sum_3\}\rightarrow\{\sum_{w5}\}$	✓	D_6-D_5	D_4	$-D_3+D_2$	0	θ_{13}	0
$\{\sum_4\}\rightarrow\{\sum_{w6}\}$	✓	D_6-D_5	$-D_4$	$-D_3+D_2$	0	θ_{14}	0

Note: x, y, z, θ_x, θ_y, θ_z: transform parameters with respect to the parent frame; θ_i represents the variable of the joint angle.

Supplementary Table 12 Mechanical properties of Apollo samples and simulants[5]

	Bulk Density ($g\ cm^{-3}$)	Particle Specific Gravity	Shear Strength	
			Cohesion c (kPa)	Friction Angle φ (°)
Lunar Soil (Averaged)	1.53 – 1.63 (Near Surface)	2.3 - 3.2	0.1 - 1	30 – 50
FJS-1	1.55	2.94	8	37.2
Lunar soil simulant[6]	1.6	-	0.8	37.2

Supplementary Table 13 The physical and mechanical parameters of the lunar soil simulant and HIT-LSS2[7, 8]

	ρ ($kg\ m^{-3}$)	k_c ($kPa\ m^{-n+1}$)	k_φ ($kPa\ m^{-n}$)	n	c (kPa)	φ (°)	K_x (m)	K_y (m)
Lunar soil simulant[6]	1.600	1.71	4754.67	1.00	0.8	37.20	$0.043\beta + 0.036$	$0.020\beta + 0.013$
HIT-LSS2[7,8]	1.600	0	1043.0	0.87	0.46	38.1	$0.005503\beta + 0.008302$	$0.045\beta + 0.01201$

Note: ρ is bulk density, k_c is cohesion modulus of the soil, k_φ is the frictional modulus of the soil, n is the intrinsic sinkage exponent of the terrain (it is deemed that $n = N$, when the dynamic sinkage caused by slip ratio is not considered), c is the cohesion of the soil, φ is the internal friction angle of the soil, K_x is the longitudinal shearing deformation modulus of the soil, K_y is the lateral shearing deformation modulus of the soil.

Supplementary References

1. Gou, S. *et al*. Impact melt breccia and surrounding regolith measured by Chang'e-4 rover. *Earth Planet. Sci. Lett.* **544**, 116378 (2020).
2. Li, C., Xu, R., Lv, G., Yuan, Y., He, Z., Wang, J. Detection and calibration characteristics of the visible and near-infrared imaging spectrometer in the Chang'E-4. *Rev. Sci. Instrum.* **90**, 103106 (2019).
3. Wong, J. Y. *Theory of Ground Vehicles* (John Wiley & Sons, New Jersey, 2008).
4. French, B. M., Heiken, G., Vaniman, D., Schmitt, H. H., & Schmitt, J. *Lunar Sourcebook A Users Guide to the Moon* (Cambridge Univ. Press, 1991).
5. Kanamori, H., Udagawa, S., Yoshida, T., Matsumoto, S., Takagi, K., Properties of lunar soil simulant manufactured in Japan. In *the 6th International Conference and Exposition on Engineering, Construction, and Operations in Space,* Albuquerque, USA, 1998.
6. Ishigami, G. Terramechanics-based analysis and control for lunar/planetary exploration robots. PhD thesis, Tohoku University (2008).
7. Li, J. Research on wheel soil interaction mechanics for planetary exploration rovers under cornering and slip conditions. Master thesis, Harbin Institute of Technology (2017).
8. Ding, L. *et al*. Experimental Study and Analysis of the Wheels' Steering Mechanics for Planetary Exploration WMRs Moving on Deformable Terrain. *Int. J. Robot. Res.* **32**, 712–743 (2013).
